# An Action-Independent Role for Midfrontal Theta Activity Prior to Error Commission

**DOI:** 10.3389/fnhum.2022.805080

**Published:** 2022-05-11

**Authors:** João Estiveira, Camila Dias, Diana Costa, João Castelhano, Miguel Castelo-Branco, Teresa Sousa

**Affiliations:** ^1^CIBIT – Coimbra Institute for Biomedical Imaging and Translational Research, University of Coimbra, Coimbra, Portugal; ^2^ICNAS – Institute for Nuclear Sciences Applied to Health, University of Coimbra, Coimbra, Portugal; ^3^FMUC – Faculty of Medicine, University of Coimbra, Coimbra, Portugal

**Keywords:** error-monitoring, performance monitoring, midfrontal theta, pre-error neuronal patterns, post-error neuronal patterns

## Abstract

Error-related electroencephalographic (EEG) signals have been widely studied concerning the human cognitive capability of differentiating between erroneous and correct actions. Midfrontal error-related negativity (ERN) and theta band oscillations are believed to underlie post-action error monitoring. However, it remains elusive how early monitoring activity is trackable and what are the pre-response brain mechanisms related to performance monitoring. Moreover, it is still unclear how task-specific parameters, such as cognitive demand or motor control, influence these processes. Here, we aimed to test pre- and post-error EEG patterns for different types of motor responses and investigate the neuronal mechanisms leading to erroneous actions. We implemented a go/no-go paradigm based on keypresses and saccades. Participants received an initial instruction about the direction of response to be given based on a facial cue and a subsequent one about the type of action to be performed based on an object cue. The paradigm was tested in 20 healthy volunteers combining EEG and eye tracking. We found significant differences in reaction time, number, and type of errors between the two actions. Saccadic responses reflected a higher number of premature responses and errors compared to the keypress ones. Nevertheless, both led to similar EEG patterns, supporting previous evidence for increased ERN amplitude and midfrontal theta power during error commission. Moreover, we found pre-error decreased theta activity independent of the type of action. Source analysis suggested different origin for such pre- and post-error neuronal patterns, matching the anterior insular cortex and the anterior cingulate cortex, respectively. This opposite pattern supports previous evidence of midfrontal theta not only as a neuronal marker of error commission but also as a predictor of action performance. Midfrontal theta, mostly associated with alert mechanisms triggering behavioral adjustments, also seems to reflect pre-response attentional mechanisms independently of the action to be performed. Our findings also add to the discussion regarding how salience network nodes interact during performance monitoring by suggesting that pre- and post-error patterns have different neuronal sources within this network.

## Introduction

Humans are constantly processing sensory information from the surrounding environment and adapting their actions accordingly. This monitoring process can be understood as a constant performance evaluation mechanism, where observed or performed action outcomes are compared against individual expectations. These continued verifications shape behavior, help avoid error repetition, and are fundamental to short- and long-term learning processes (Ullsperger et al., 2014). While the neuronal mechanisms underlying post-action performance monitoring are relatively well-identified and documented within the scientific literature, the pre-action ones are still not fully understood. It is commonly accepted that pre-action mental states play a major role in performance, but the neuronal patterns underlying key mechanisms to success remain less studied.

Performance monitoring neuronal processes are widely investigated using electroencephalography (EEG) through several event-related potentials (ERPs) – as the error-related negativity (ERN), correct-related negativity (CRN), and error-related positivity (Pe) – and midfrontal theta. Such performance monitoring neuronal markers have been related to behavior adaptation (Cohen, 2011), executive functioning (Mohamed et al., 2019), subjective emotional feeling (Dan Glauser and Scherer, 2008), and attentional control (Cavanagh et al., 2012). Moreover, some of them are believed to be altered in several neuropsychiatric disorders, including depression, schizophrenia, and autism (Olvet and Hajcak, 2008; Bates et al., 2009; Santesso et al., 2011).

The ERN and CRN components, the most studied performance monitoring EEG patterns, can be recorded at the midfrontal channels at approximately 50–100 ms after an erroneous or correct response, respectively (Falkenstein et al., 2000). ERN is a reliable error-processing index, likely reflecting activity related to the ongoing evaluation of errors and response conflict and functioning as an error signal at the remedial action system (Gehring et al., 2012). Nevertheless, there is increasing evidence that ERN also relates to motivational and affective variables and might be tied to neuronal mechanisms supporting defensive behaviors and avoidance learning (Olvet and Hajcak, 2008). In addition, a small ERN-like component is sometimes evident on correct response trials – the CRN component. It has been suggested that CRN reflects a response comparison process, and higher CRN amplitude has been found to reflect task engagement (Simons, 2010).

The anterior cingulate cortex (ACC) has been pointed out by EEG and functional magnetic resonance imaging (fMRI) studies as the ERN and CRN neuronal source (Iannaccone et al., 2015; Pavone et al., 2016). Several theories and computational models have been developed regarding the error-monitoring processes, but, in brief, it is commonly accepted that the ACC signals the prefrontal cortex that an increase in attention or cognitive control is required (Orr and Hester, 2012).

The Pe component has been also described, particularly in the centroparietal electrodes, between 200 and 400 ms after erroneous responses (Martin et al., 2018). It has been suggested that its amplitude correlates with confidence in perceptual decisions and general quality of the metacognitive decision process (Boldt and Yeung, 2015). This later ERP might discriminate between detected and undetected errors, but there is still no consensus regarding its precise functional significance.

In addition to the error-related components, midfrontal theta has assumed a relevant role as a neuronal marker of error monitoring processes (Cavanagh et al., 2012; Cavanagh and Frank, 2014; Pavone et al., 2016). Midfrontal theta activity has been linked to several complex mechanisms such as focused attention, information encoding, cognitive load, and response control (Cavanagh and Frank, 2014). Moreover, theta power enhancement is believed to be a reliable measure of cognitive control recruitment across different types of conflicts in the stream of information processing (Nigbur et al., 2011). This is also in line with evidence for increased theta power during attention-demanding tasks found to be inversely related to decreased default mode network activity (Scheeringa, 2008). Such ubiquity of midfrontal theta has been suggested to signal a generic processing mechanism for coordinating endogenous and exogenous performance-relevant information (Cavanagh et al., 2012) and to reflect a common neuronal computation used for realizing the need for cognitive control and its communication across disparate brain regions (Cavanagh and Frank, 2014).

Its increased power during error commission is, therefore, thought to result from the medial prefrontal cortex signaling for enhanced control (Cavanagh et al., 2012; Cavanagh and Frank, 2014) and to be related to behavioral adaptation in reinforcement learning (Cavanagh et al., 2010), particularly in feedback learning (Kaiser et al., 2021). It has also been reported that the ERN is, in part, a result of the ongoing theta modulation that occurs following erroneous actions (Trujillo and Allen, 2007; Cohen et al., 2008), highlighting the importance of the midfrontal theta in error-related processes.

Some studies have described midfrontal theta power modulation already during response preparation and as a predictor of success (Cavanagh et al., 2009; Ruiz et al., 2011; van Noordt et al., 2017; Gomez-Pilar et al., 2018; Dias et al., 2022). Nevertheless, there is no agreement regarding which direction such activity modulation occurs and its functional meaning. Increased theta power during response preparation has been related to information prioritization (Wallis et al., 2015; de Vries et al., 2020), memory encoding (White et al., 2013), and coordination of neural processing in the sensorimotor pathways of the brain to support efficient decision-making (Cohen and Donner, 2013). Moreover, recruiting executive control to resolve upcoming behavioral challenges has been linked to modulation of theta activity in medial frontal neurons, both in studies with non-human and human samples (Totah et al., 2013; van Noordt et al., 2017). It has also been suggested that theta power decrease prior to error commission might highlight mind-wandering states (Atchley et al., 2017) and mechanisms of communication between action monitoring and cognitive control networks (Cavanagh et al., 2009).

Recent studies have found evidence for distinct midfrontal theta inputs (Töllner et al., 2017; Zuure et al., 2020), thus suggesting rather than a unitary neural mechanism of cognitive control (Cavanagh et al., 2012; Cavanagh and Frank, 2014; Kaiser et al., 2019; Duprez et al., 2020), it might reflect different underlying neural processes depending on the task context. In line with this, Kaiser and Schütz-Bosbach (2021) found that pre-response theta is linked to synchronization of task-relevant brain areas and demonstrated domain-specific effects of theta power and connectivity. Their findings highlighted the need for further dissociation between general and domain-specific neural effects during different types of behavioral interactions as an important step for the understanding of the midfrontal theta role neural in cognitive control.

Here, we wanted to study the neuronal mechanisms underlying performance monitoring, both during response preparation and execution, based on two different types of actions/responses and using a go/no-go task. We tested both oculomotor and motor responses to clarify how error-monitoring neuronal mechanisms vary according to the action performed. Moreover, we followed the hypothesis of midfrontal theta reflecting not only the reactive cognitive control required during the adaptive processes after error commission but also the one required during response planning (van Noordt et al., 2017). The go/no-go task required fast attentional engagement/disengagement and the two types of responses tested entailed different levels of complexity.

Error-related neuronal patterns have been studied using different tasks, but the influence of task-specific parameters such as cognitive demand or motor control remains elusive. It is believed that when visual information is available, hand and eye movements are generated independently of each other (Frens and Erkelens, 1991). Saccades use only visual information, while hand movements processes use visual as well as cognitive information. Therefore, saccades tend to be more automatic than hand movements. We thus hypothesized eye movements to be more automatic and to result more often from impulsive reactions to instantaneous visual stimuli, while hand movements are expected to more often be under volitional control. To scrutinize which neural performance monitoring processes are truly invariant irrespective of the type of action, we performed a comparative ERN and theta analysis in the moments preceding and following each type of action. Our main goal was to test error-related components and midfrontal theta modulation elicited by error commission and theta patterns predictive of success, irrespective of the type of action.

## Experimental Methodology

We designed a go/no-go paradigm involving two different motor tasks (hand-motor and oculomotor) to evaluate behavioral performance and self-monitoring EEG signals associated with different types of action. keypresses, eye movements (namely saccadic events), and EEG were recorded simultaneously during the experimental procedure. Behavioral data allowed for determining the type of committed errors and reaction time evaluation whereas, EEG was used to map ERPs and theta modulation signatures related to performance monitoring, as well as to estimate the neuronal sources of such patterns.

### Participants

Twenty healthy volunteers (25.55 ± 3.72 years, 10 male) participated in our study. All participants except one were right-handed as confirmed by a handedness questionnaire adapted from Oldfield (1971): mean laterality index of 80.25 ± 20.14. All had a normal or corrected-to-normal vision, and none of them reported neuropsychiatric disorders. All provided written informed consent in accordance with the Declaration of Helsinki prior to participation. The study followed the safety guidelines for human research and was approved by the Ethics Committee of the Faculty of Medicine of the University of Coimbra.

One participant (P3) was excluded from the keypress analysis due to the absence of erroneous responses, and another one (P7) was excluded from the pre-response analysis due to excessive interference of ocular artifacts.

### Go/No-Go Paradigm

**FIGURE 1:**
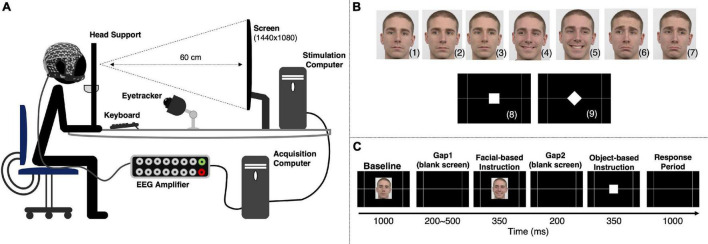
Experimental setup and stimulation paradigm. **(A)** Experimental setup used to simultaneously acquire behavioral and EEG data. **(B)** The go/no-go paradigm included two types of instruction. A facial cue provided information about the response direction and was based on three facial expressions: neutral [no-go to the left (2) or right (3)], happy [pro-keypress/saccade to the left (4) or right (5)], and sad [anti-keypress/saccade to the left (6) or right (7)]. An object cue indicated the type of response to be given [keypress (8) or saccade (9)]. Successful responses depended upon the correct interpretation of the two instructions. The facial images are from Radboud Faces Database (Langner et al., 2010). **(C)** Representation of an experimental trial. An experimental run comprised 120 trials, with each participant completing 4 runs. All the instructions were presented the same number of times per run.

The participants were comfortably sitting 60 cm away from the stimulation screen (Cambridge Research Systems Display++, 32 inches LCD Monitor using a 1440 × 1080 pixel resolution and a 60 Hz refresh rate, connected to the stimulation computer with 16 GB of RAM, Intel^®^ Xeon^®^ CPU E3-1270 v5 @ 3.60 GHz processor running 64-bit Windows 10 Pro), resting the head in the eye-tracking head support, and holding a steady right hand above the left and right arrows of the keyboard (Figure 1A).

The stimulation sequence was based on two primary instructions: a facial cue and an object cue. One of the three facial expressions – neutral, happy, or sad – indicated either not act, act in the same direction of the face’s eye gaze, or in the opposite one, respectively. Therefore, combining facial expressions and gaze direction resulted in six different facial instructions (Figure 1B): neutral-left no-go (2), neutral-right no-go (3), happy-left pro-action (4), happy-right pro-action (5), sad-left anti-action (6) and sad-right anti-action (7). The object-based cues (Figure 1B) – a white square (8) or a white diamond (9) – instructed participants to either perform a keypress or a saccade, respectively. A correct response required a correct interpretation of both instructions.

The experimental paradigm included six sequential periods (Figure 1C). First, participants were presented with a neutral face (direct gaze) for 1000 ms (*Baseline*). It was followed by a blank screen (*Gap1*) randomly varying between 200 and 500 ms to avoid anticipation. Then, one of the six available facial-based instructions was randomly presented for 350 ms and followed by another blank screen period (*Gap2*) of 200 ms. Next, an object-based instruction was randomly selected and presented for 350 ms. Finally, participants had 1000 ms to give a response (*Response*). Participant’s responses recorded during the object-based instruction period were considered as premature responses.

Facial instruction images had a vertical visual angle of 9.02° and a horizontal visual angle of 6.79° (from center to periphery). The square and diamond presented during the object-based instruction had a vertical visual angle of 1.82° and 2.57°, and a horizontal visual angle of 1.82° and 2.57°, respectively (from center to periphery). A gray grid designed to help homogenize saccade behavior possessed intersections at a horizontal visual angle of 14.12° (measured from the center of the screen). The stimulation sequence was designed using Psychophysics Toolbox 3.0 for MATLAB R2019b. The images used for the facial-based instruction (Figure 1B) are from a single male Caucasian individual, obtained from Radboud Faces Database (Langner et al., 2010).

Each participant performed 4 experimental runs, each run comprising 120 trials (40 no-go, 20 pro-keypress, 20 pro-saccade, 20 anti-keypress, and 20 anti-saccade). Each run took approximately 7 min, including a two-step eye-tracker calibration.

### Data Recording: EEG, Keypress, and Eye-Tracking

EEG was recorded from 64 channels using an extended international 10–20 system (QuickCap from Neuroscan, United States, SynAmps 2 amplifier from Compumedics NeuroScan, United States and Curry NeuroImage 7.08 software from NeuroScan, United States), with a 500 Hz sampling rate and a monopolar montage using a ground electrode near the Cz channel. Electrooculogram (EOG) was recorded with a 500 Hz sampling rate and a bipolar montage (VEOU and VEOD electrodes positioned above and below the left eye, respectively, and HEOL and HEOR electrodes positioned on the outer side of the left and right eyes, respectively). EEG impedances were kept below 20 kΩ as much as possible.

Eye-tracking data were recorded using the left eye (EyeLink 1000 Plus from SR Research, Canada). Each run started with an eye-tracker 9-point calibration session, followed by a 9-point validation session. Eye-tracker was recorded with a 1000 Hz sampling rate and a 0.25° 0.50° accuracy. Keypress and saccadic trial information, namely movement direction, response latency, and performance evaluation data were registered using MATLAB files (*.mat).

### Response Identification

**FIGURE 2:**
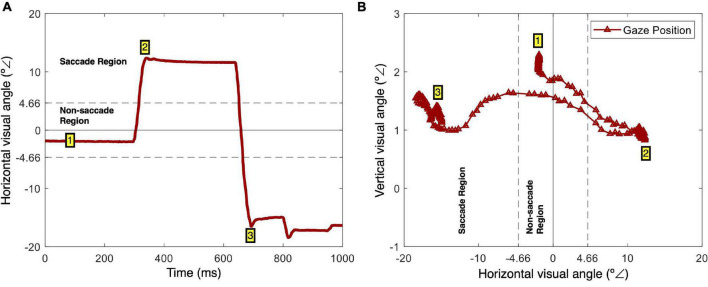
Saccade identification. **(A)** Example of horizontal gaze position (*x* coordinates in visual angles) as a function of time. There is an initial fixation (1), followed by a right-side saccade (2), and a correction left-side saccade (3). **(B)** Screen gaze positions (*x* and *y* coordinates in visual angles) during the same period. This is an alternative representation of the gaze path, with an initial fixation (1), a right-side saccade (2), and a correction left-side saccade.

Figure 2 schematically represents the saccadic identification method used. Micro-saccades, often defined as involuntary eye movements that occur during fixation, are a phenomenon whose definition is still somewhat controversial, suggesting that definitions commonly depend on the specific experimental task (Poletti and Rucci, 2016). Here, saccades were defined as horizontal movements superior to 4.66° from the screen center. Figure 2A illustrates the horizontal visual angle as a function of time for a trial in which two saccades were performed. Figure 2B illustrates the gaze positions (vertical and horizontal visual angles) from this trial. In this example, after the initial fixation on the screen center (1), a first saccade to the right side of the screen occurred (2). The saccade start was defined as the point where the horizontal gaze velocity reached zero after moving from the screen center to the periphery (Gibaldi and Sabatini, 2021). Then a second saccade occurred – the gaze shifted from the right side to the left side of the screen (3). This saccadic identification method was based on the instruction given to the participants – they were asked to fixate the center of the screen and follow the grid presented during stimulation when a saccadic movement was required to standardize such response as much as possible (Figure 1C). Regarding the keypress responses, the action was considered as soon as a key was pressed.

### Behavioral Data Analysis

To evaluate participants’ performance, we analyzed how well their responses followed the facial-based and object-based cues. Moreover, errors during keypress and saccade trials were analyzed separately due to the different nature of both actions (Frens and Erkelens, 1991).

Errors related to the facial-based instruction were divided into three types of commission errors – *Go instead of No-go* (performing the corresponding action instead of no action), *Anti instead of Pro* (performing the corresponding action in the opposite direction of the facial-based instruction gaze, when it should have been the opposite), and *Pro instead of Anti* (performing the corresponding action in the same direction as the facial instruction gaze, when it should have been the opposite) – and one type of omission errors – *No-go instead of Go* (no action instead of performing the corresponding action). Errors related to the facial-based instructions were only evaluated for the corresponding action of those specific trials. Thus, in square instruction trials were only evaluated keypress actions, and in diamond instruction trials were only evaluated saccadic actions.

Errors related to the object-based instruction were divided into four types of commission errors: *Both instead of Keypress* (both actions performed in a keypress trial), *Both instead of Saccade* (both actions performed in a saccade trial), *Saccade instead of Keypress* (a saccade performed instead of a keypress), and *Keypress instead of Saccade* (a keypress performed instead of a saccade). Errors related to the object-based instruction were evaluated for all trials. If for a given trial an object-based instruction error coincided with a facial-based instruction correct response, then that trial was removed from facial-based instruction sample of correct responses.

Within the errors regarding facial-based instruction, we calculated the relative frequency of reaction time by counting the number of committed errors in time intervals of 50 ms (20 intervals from 0.05 to 1 s) and dividing the result of each interval by the total number of events of that type. Premature responses (responses given before the defined period) were not included in this calculation. We included in this analysis erroneous and correct keypress, and erroneous and correct saccades.

### EEG Data Processing

EEG data were processed and analyzed using a MATLAB R2021b script based on the EEGLAB toolbox (14.1.2b). Data were filtered using a Hamming windowed *sinc* finite impulse response (FIR) filter between 0.5 and 100 Hz. Required filter order was estimated with the following heuristic in default mode: transition band width is 25% of the lower passband edge (not lower than 2 Hz). A notch filter was applied between 47.50 and 52.50 Hz. Noisy channels were identified, removed, and interpolated using the spherical linear interpolation method. Data were then re-referenced to the average (EOG channels were excluded from this step) of all channels.

Finally, we ran independent component analysis (ICA) to minimize artifacts embedded in the EEG data (muscle, eye blinks, or eye-movements) without removing the affected data portions (Keren et al., 2010; Delorme et al., 2012; Castelhano et al., 2014; Sousa et al., 2017; Dimigen, 2020). We used Infomax algorithm of EEGLAB toolbox (implemented in *runica.m*), which is based on Tony Bell’s infomax algorithm as implemented for automated use by Makeig et al. (1996) using the natural gradient of Amari (1999). We applied the extended option suggested by Lee et al. (2000) to also extract sub-Gaussian sources. ICA components were then visually inspected and removed. After pre-processing, the individual datasets were segmented as detailed below.

#### Post-response Data

Correct vs. error trials were analyzed for post-response moments through response-locked epochs. A correct sample required successful actions based on both facial and object instruction. Premature but correct responses were included. The last 200 ms of the *Gap1* (blank screen) period (Figure 1C) were used as a baseline. Epochs included data from −2200 to 500 ms and were centered on the beginning of response (moment defined as described in section “Response Identification”). An algorithm was used to calculate the latency difference between response and baseline, individually subtracting the EEG baseline from every trial. Due to the absence of action in omission (*No-go instead of Go*) errors related to the facial-based instruction, this type of error was not included in this analysis.

#### Pre-response Data

In the case of the pre-response analysis, correct samples required successful actions based on both facial and object instruction. Premature responses were not included to prevent contamination of pre-response signals with post-response signals. Epochs were centered on the beginning of *Response* period (Figure 1C) and the epoch length set from −1200 to 100 ms. The last 200 ms of the *Gap1* (blank screen) period [−1100 −900] ms, were used as the baseline.

### EEG Data Analysis

#### Event-Related Potentials

We analyzed the ERN latency and amplitude for channels Fz, F1, F2, FCz, FC1, and FC2 (Dan Glauser and Scherer, 2008; Cohen, 2011; Cavanagh et al., 2012; Mohamed et al., 2019). The minimum peak in the [0 100] ms interval, where the ERN is reported to be elicited (Falkenstein et al., 2000), was selected. Keypress and saccade trials were analyzed separately.

#### Midfrontal Theta Power

Theta frequency analysis was run for the pre- and post-response moments by measuring the power spectral density (PSD). The analysis encompassed frequencies in the range of 4–7 Hz, previously described to be associated with error monitoring processes (Cavanagh et al., 2012; Cavanagh and Frank, 2014; Pavone et al., 2016). For performance related to facial-based instruction (instructing direction of action), the post-response analysis was response-locked in the [0 200] and [100 300] ms intervals (intervals centered in the PSD peak for each type o response – keypress and saccade, respectively). For performance related to object-based instruction (indicating type of action), the post-response analysis considered the interval from [0 300] ms to account for higher response latency variability, as this analysis encompasses responses based on both types of action – keypress and saccades.

For performance related to facial-based instruction, the pre-response analysis was centered in the response preparation period immediately before response execution [−300 −100] ms, where attentional levels have been hypothesized to be related to theta modulation (Cavanagh et al., 2009; Labrenz et al., 2012; Chmielewski et al., 2014; Atchley et al., 2017; Pscherer et al., 2019, 2020). Time-length was defined to match the post-response analysis and avoid contamination with response potentials. Two additional pre-response periods were considered as exploratory analysis, namely [−700 −500] ms (matching the period after the facial instruction) and [−500 −350] ms (matching the blank screen presented between instructions). For performance related to object-based instruction, we considered a slightly larger pre-response period [−350 −100] ms, to account for higher variability of error-related processes.

#### Source Analysis

Based on scalp-recorded electrical potential distribution, the standardized low-resolution electromagnetic tomography method (sLORETA) – implemented by sLORETA-KEY (v20201109) software – was used to compute the cortical three-dimensional (3D) distribution of current density (Pascual-Marqui et al., 1994; Pascual-Marqui, 2002). The sLORETA method is a standardized discrete, 3D distributed, linear, minimum norm inverse solution. The employed standardization endows the tomography with the property of exact localization to test point sources, yielding images of standardized current density (SCD) with exact localization, albeit with a low spatial resolution (Pascual-Marqui, 2009). The analysis focused on the theta power for pre- and post-response moments, and on the ERPs (post-response), was run separately for keypresses and saccades and considering performance related to facial-based instruction. The neuronal sources were estimated based on SCD maps following the Montreal Neurological Institute (MNI) coordinate system and the SCD peak coordinates. The brain regions best matching such peak coordinates were identified as an approximation.

#### Statistical Analysis

EEG and behavioral statistical analyses were performed using IBM SPSS Statistics 27 software. The statistical analysis was based on linear mixed-effects modeling to account for the several factors we were interested in while taking into account an unequal number of repetitions, within and between participants, of errors and correct samples. The fixed effects were considered as our factors of interest and the random ones the inter-subject variability.

Behavioral statistical analyses were conducted over reaction time to test whether it depended on performance (correct and error) and type of response (keypress and saccade) and if there was a significant interaction between both factors.

Statistical analyses of the neuronal patterns of errors related to facial-based instructions were conducted over the F1, Fz, F2, FC1, FCz, and FC2 channels. ERN amplitude, here estimated as the minimum peak value, and latency (post-response), and theta modulation in pre- and post-response moments were tested. For ERN amplitude statistical analysis, the type of response (keypress or saccade) and performance (correct or error) were considered, as well as the interaction between both factors. ERN latency was tested for possible differences depending on the type of response. Additionally, the ERN/CRN modulation during errors related to object-based instructions was also tested. In this case, as the erroneous trials included mixed types of responses, the response type was not considered as a factor of interest.

Theta statistical analysis was also separately run for performance related to facial-based and object-based instructions due to different definitions of error for each case. For performance related to facial-based instructions, the midfrontal theta was statistically tested for differences depending on performance (correct or error), type of response (keypress or saccade), and time (pre- and post-response), and for interactions between all factors (performance × action, performance × time, action × time). For performance related to object-based instructions, the midfrontal theta was statistically tested for differences depending on performance (correct or error), and time (pre- and post-response), and for interactions between both factors.

Source statistical analyses were performed in the sLORETA-KEY software interface and using dependent groups analysis (paired *t*-test). Sources were estimated for the minimum *p*-value.

All tests were performed with a 95% confidence interval and the results were considered significant for *p* < 0.05.

## Results

### Behavioral Data

Considering all 9600 trials (480 trials *per* participant), we found that the errors related to the facial-based instruction (*n* = 882 errors) represented 9.19% of the responses, and the errors related to the object-based instruction (*n* = 1043 errors) represented 10.86%. When considering each type of response separately (4800 trials each), keypress trials resulted in an error rate related to facial-based instruction of 7.19% (*n* = 345 errors) and an error rate related to object-based instruction of 19.67% (*n* = 944 errors). Saccade trials resulted in an error rate to facial-based instruction of 11.18% (*n* = 537 errors) and an error rate related to object-based instruction of 2.06% (*n* = 99 errors).

**FIGURE 3:**
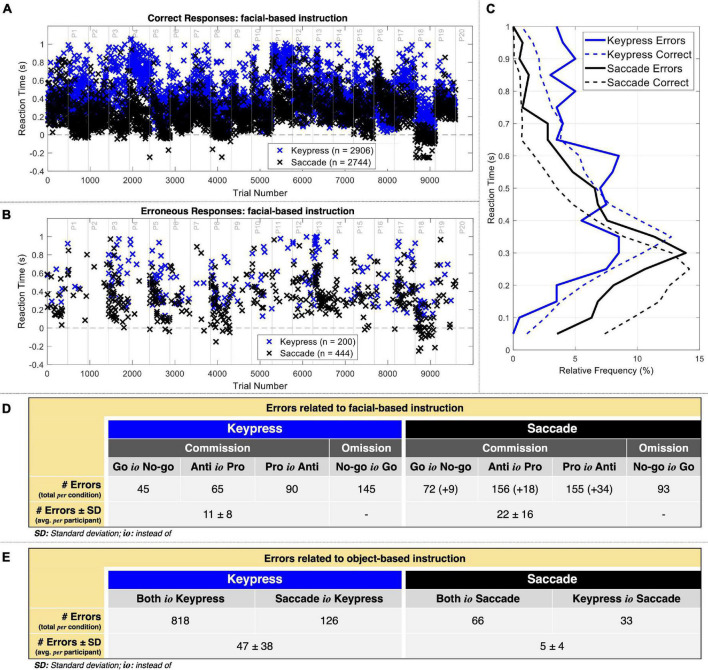
Behavioral data. **(A)** Reaction time of correct responses when considering the facial-based instruction. Correct premature responses occurred mostly in saccades. **(B)** Reaction time of commission errors when considering the facial-based instruction. Again, there were several premature responses in saccades. **(C)** Relative frequency (%) of reaction time for 50 ms intervals. **(D)** Number of errors related to the facial-based instruction for keypress and saccade trials. The numbers in curved parenthesis represent the erroneous premature responses included in the EEG post-response analysis. The average omission errors *per* participant were not calculated given that these were not used for EEG analysis. **(E)** Number of errors related to the object-based instruction for keypress and saccade trials.

Saccadic responses led to more errors and tended to be given quicker than keypresses (Figure 3). Considering the facial-based instruction, keypress revealed an average reaction time of 532.7 ± 237.7 and 445.3 ± 212.9 ms for erroneous and correct responses, respectively. Saccades revealed an average reaction time of 348.7 ± 107.8 and 270.9 ± 163.8 ms for erroneous and correct responses, respectively. The statistical analysis revealed a significant interaction between performance and type of response given contributing to the reaction time [*F*(*df*) = 4.02 (5954.14), *p* = 0.04]. We then analyzed each type of response separately and found that reaction time was 116.57 ± 12.87 ms shorter for correct than erroneous keypresses responses [*t*(*df*) = −9.05 (3080.65), *p* = 2.41 × 10^–19^]. Similarly, we found that reaction time was 92.21 ± 8.47 ms shorter for correct than erroneous saccade responses [*t*(*df*) = −10.76 (2876.69), *p* = 1.63 × 10^–26^). The relative frequency of reaction time of keypress responses peaked at the [300 350] ms interval for correct while presented three peaks for erroneous responses at the [200 300], [300 350], and [550 600] ms intervals (Figure 3C). Saccade correct responses peaked at the [200 250] ms interval and the erroneous at the [250 300] ms interval. These reaction time results did not take into account premature responses.

Figure 3D presents the distribution of errors related to facial-based instruction. Omission errors (*No-go instead of Go*) represented 42.03% of keypress errors (*n* = 145) and 17.31% of saccade (*n* = 93) errors. Regarding commission errors, *Go instead of No-go* actions represented 13.04% of keypress (*n* = 45) and 15.08% of saccade (*n* = 81) errors, *Anti instead of Pro* actions represented 18.84% of keypress (*n* = 65) and 32.40% of saccade (*n* = 174) errors, and *Pro instead of Anti actions* represented 26.09% of keypress (*n* = 90) and 35.20% of saccade (*n* = 189) errors, respectively. On average, we thus considered for neurophysiological analysis of the errors related to facial-based instruction 11 ± 8 and 22 ± 16 keypress and saccade commission errors per participant, respectively.

Figure 3E represents the distribution of errors related to object-based instruction. This analysis included only commission errors. *Both instead of Keypress* responses represented 78.42% (*n* = 818) of all object-related errors against the 6.33% resulting from *Both instead of Saccade* responses (*n* = 66). Also, *Saccade instead of Keypress* responses represented 12.08% (*n* = 126) of all object-related errors against the 3.16% resulting from *Keypress instead of Saccade* responses (*n* = 33). On average, we thus considered for neurophysiological analysis of the errors related to object-based instruction 47 ± 38 and 5 ± 4 keypress and saccade commission errors per participant, respectively.

The accounted saccadic movements, as identified by our saccade detection algorithm, revealed standard velocity values: average velocity of 406.30 ± 37.25°/s (Raab, 1985) and average maximum instantaneous velocity of 799.94 ± 113.26°/s (Leigh and Zee, 2015; Lemos, 2016).

### Neuronal Patterns Underlying Errors Related to the Facial-Based Instruction

#### Error-Related Negativity and Correct-Related Negativity

**FIGURE 4:**
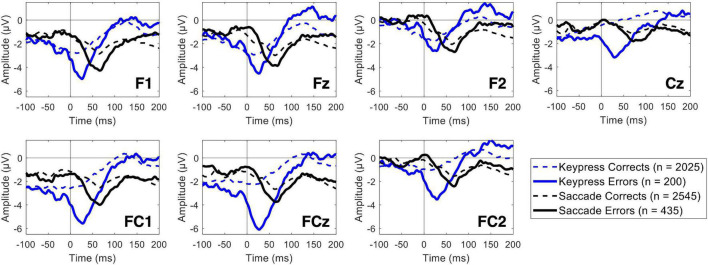
Event-related potentials for commission errors (ERN) and correct responses (CRN) over the midfrontal channels, including the Cz channel. The blue lines represent the keypress actions, while the black lines represent the saccadic actions. The solid and dashed lines represent erroneous and correct responses, respectively.

Both keypress and saccade erroneous responses elicited evident ERN modulation (Figure 4). The ERN average latency recorded in the FCz channel was 41.95 ± 28.95 and 59.3 ± 30.49 ms for keypress and saccade errors, respectively. It revealed to significantly vary depending on the response type [*F*(*df*) = 46.54 (620.53), *p* = 2.14 × 10^–11^), being the ERN latency 17.39 ± 2.55 ms shorter for keypress than saccades [*t*(*df*) = −6.82 (620.53)]. Such latency difference was observed for all analyzed channels. For simplification, we present here the results for the FCz channel, but all the results are detailed in Supplementary Table 1.

The ERN minimum peak value revealed to be significantly lower during both keypress and saccade erroneous responses than during the correct ones. Nevertheless, a significant interaction between the type of response and the ERN/CRN amplitude modulation by the participants performance was found for some channels, as it was the case of FCz [*F*(*df*) = 7.14 (4883.82), *p* = 0.008]. By analyzing each type of response separately, we found that the ERN/CRN minimum peak value was 2.49 ± 0.43 μV more negative for erroneous than corrects keypresses [*t*(*df*) = 5.85 (1966.40), *p* = 5.92 × 10^–9^]. Similarly, the ERN/CRN minimum peak value was 0.91 ± 0.29 μV more negative for erroneous than corrects saccades [*t*(*df*) = 3.19 (2816.94), *p* = 0.001]. This interaction was only found for FCz and FC2 channels, as detailed in Supplementary Table 2. The fixed effects estimates are detailed in Supplementary Table 3.

#### Pre- vs. Post-error Midfrontal Theta

**FIGURE 5:**
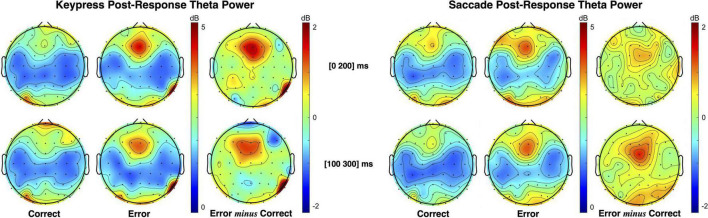
Topographic maps of theta (4–7 Hz) power spectral density (PSD) distribution following keypress and saccadic actions. The maps illustrate that theta power increase occurs in different time intervals for keypress and saccade (as seen in the ERN). Statistical analysis was performed in the [0 200] ms interval for keypress responses and in the [100 300] ms interval for saccade responses, where the PSD of each response occurs, albeit both intervals are here presented for illustrative purposes.

Midfrontal theta modulation was analyzed for pre- and post-response moments, depending on the obtained performance and type of responses used. Post-response theta power topographic maps for keypress and saccade responses are presented in Figure 5. As illustrated, the midfrontal theta power was higher for erroneous than correct responses, both for keypress and saccades. However, error-related theta modulation was most evident at different time intervals for each type of response, as expected from the differences recorded in ERN latency. Post-response theta modulation was, thus, tested considering the [0 200] ms interval for keypresses and the [100 300] ms interval for saccades (intervals centered in the PSD peak for each type o response).

**FIGURE 6:**
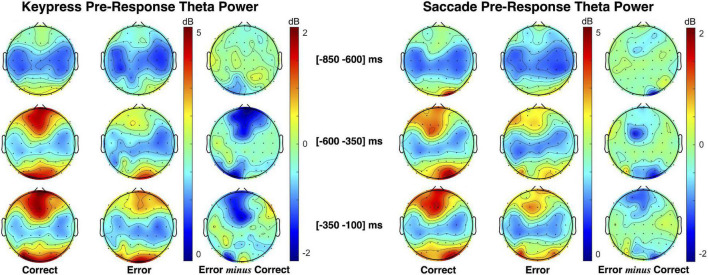
Topographic maps of theta (4–7 Hz) power spectral density (PSD) distribution during keypress **(left)** and saccadic **(right)** response preparation. The theta power was estimated centered on three moments: the facial-based instruction ([−700 −500] ms), the *Gap2* (blank screen [−500 −300] ms), and the object-based instruction ([−300 −100] ms).

Pre-response theta power patterns for keypress and saccade trials are presented in Figure 6. As illustrated by the theta power topographic maps, pre-response theta modulation is opposite to the one verified during post-response moments. Here, we found lower midfrontal theta power when preceding erroneous than correct responses. Moreover, the theta patterns verified during saccade and keypress response preparation are similarly distributed over time. Midfrontal theta modulation was most evident in the interval from −300 to −100 ms immediately preceding response execution.

**FIGURE 7:**
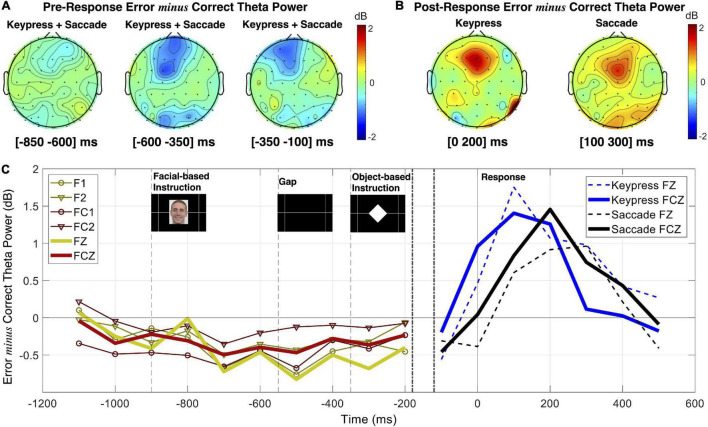
Frequency analysis comparison between response preparation and execution. **(A)** Topographic maps of theta (4–7 Hz) power spectral density (PSD) are presented as error *minus* correct events in different pre-response intervals. **(B)** Topographic maps of theta PSD for error *minus* correct events in post-response moments. **(C)** Error *minus* correct theta PSD evolution in 200 ms intervals for pre- and post-response (left and right panels, respectively).

A pre- vs. post-response comprehensive illustration is represented in Figure 7. Pre-response intervals revealed a continuously increasing error *minus* correct theta power difference for the combination of keypress and saccade trials. Over response preparation time midfrontal theta power tended to be lower when preceding incorrect responses (Figure 7A). In opposite to this, post-response midfrontal theta tended to be superior during erroneous than correct actions (Figure 7B). Figure 7C demonstrates the opposite theta modulation pattern in pre- and post-response moments, represented by the error *minus* correct theta PSD in intervals of 200 ms (baseline in the [−1100 −900] ms interval).

The statistical analysis ran based on linear mixed-effects modeling revealed an interaction between response performance (correct and erroneous) and time interval (pre- and post-) having a significant effect on midfrontal theta power modulation for the FCz channel [*F*(*df*) = 20.61 (9414.20), *p* = 5.70 × 10^–6^). Moreover, no interaction was found neither between response performance and type, nor between response type and time. Also, the statistical results revealed no significant effect of response type in theta power modulation. Thus, we did not find evidence for any further influence of the tested response types in midfrontal theta modulation during performance monitoring. We then analyzed each time interval separately for performance effects on midfrontal theta. We found that theta power was 0.69 ± 0.30 dB higher for pre-correct than pre-erroneous responses [*t*(*df*) = 2.29 (4034.89), *p* = 0.02]. In opposite to this, we found that theta power was 0.97 ± 0.22 dB lower for post-correct than post-erroneous responses [*t*(*df*) = −4.52 (4899.50), *p* = 6.27 × 10^–6^). For simplification, we present here the results for the FCz channel, but all the results are detailed in Supplementary Tables 4, 5.

### Neuronal Patterns Underlying Errors Related to the Object-Based Instruction

**FIGURE 8:**
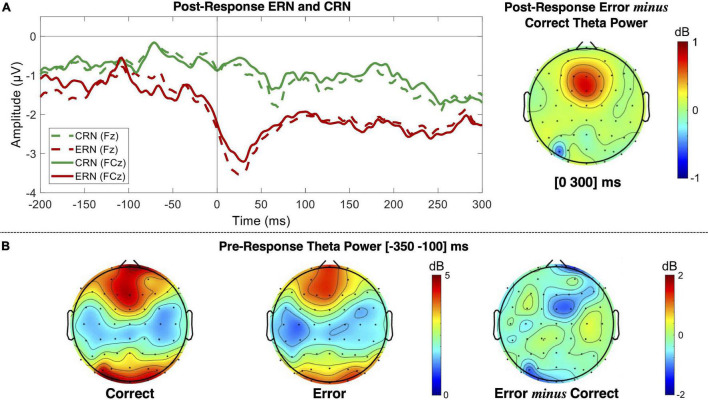
EEG patterns for performance monitoring related to the object-based instruction. **(A)** ERPs for correct (CRN) and commission erroneous (ERN) responses over the Fz and FCz channels, and post-response error *minus* correct theta power measured in the [0 300] ms interval. **(B)** Pre-response theta power for correct and erroneous trials measured in the [−350 −100] ms interval, which is stimulus-locked to the object-based instruction.

We also tested the performance monitoring patterns related to the given object-based instruction (errors where both responses were performed simultaneously or the type of response used, keypress or saccade, was swapped). The ERN/CRN and midfrontal theta modulation (pre- and post-response) are presented in Figure 8. Albeit we, here, include a high number of erroneous responses and variability of error mechanisms (different types of response separately or simultaneously given) the ERN component was evident with a minimum peak value 1.20 ± 0.26 μV lower than CRN [*t*(*df*) = 4.66 (4828.81), *p* = 3.28 × 10^–6^], as illustrated in Figure 8A.

Similar to the theta patterns revealed by error monitoring related to the facial-based instruction, here, we also found pre- and post-response opposite theta patterns depending on performance (Figures 8B). We found decreased theta power before error commission and increased theta power after error commission. The statistical analysis revealed a significant interaction between performance (correct and erroneous) and time interval (pre- and post-) impacting theta power modulation for the FCz channel [*F*(*df*) = 13.26 (8246.65), *p* = 2.72 × 10^–4^)]. We have thus analyzed each time interval separately for performance effects on midfrontal theta and found that theta power was 1.01 ± 0.45 dB higher for pre-correct than pre-erroneous responses [*t*(*df*) = 2.22 (2818.72), *p* = 0.03], and 0.49 ± 0.06 dB lower for post-correct than post-erroneous responses [*t*(*df*) = −7.87 (4626.07), *p* = 4.31 × 10^–15^]. For simplification, we present here the results for the FCz channel, but all the results are detailed in Supplementary Tables 6.

### Neuronal Sources Estimation for Pre- and Post-response Error-Related Patterns

**FIGURE 9:**
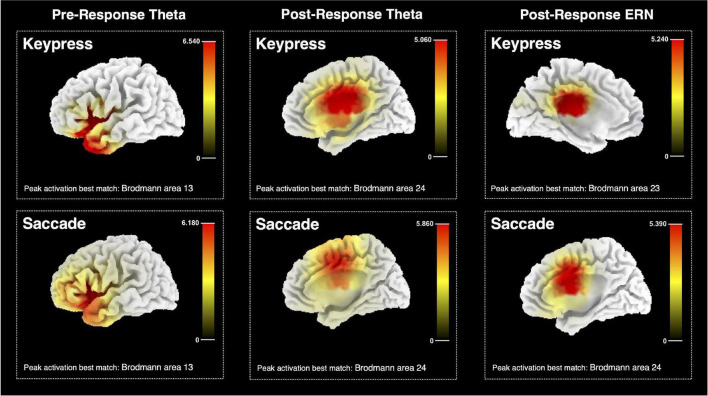
Estimated sources for pre-response and post-response midfrontal theta and ERN for keypresses and saccadic responses. The color bar represents the current source density computed with the sLORETA algorithm, with the maximum value corresponding to the peak activation. The estimated best match between the peak MNI coordinates and the brain Brodmann area is also presented.

Sources estimated in pre- and post-response moments for a keypress and saccadic actions are represented in Figure 9. The analysis was run on the theta power for pre- and post-response moments and on the ERPs (post-response), considering performance monitoring related to facial-based instructions. SCD maps were estimated and the MNI coordinates of its activation peak were identified. The best match between each identified SCD peak and a Brodmann area is also provided as an approach to the brain region estimated as the neuronal source of each tested EEG pattern. All results were estimated when thresholding the SCD maps to *p* < 0.001.

Pre-keypress theta source analysis indicated the {*X* = −35; *Y* = 15; *Z* = 0} MNI coordinates as its maximum PSD peak, which is estimated to match the Brodmann area 13, part of the insular cortex. Similar results were found for pre-saccade source analysis, which estimated the resulting PSD map to peak at {*X* = −30; *Y* = 25; *Z* = 0}, matching the same brain region. The resulting PSD map for keypress post-response theta activity was estimated to peak at {*X* = 5; *Y* = −10; *Z* = 30} MNI coordinates, approximated to the Brodmann area 24, thus possibly part of the ACC. Post-saccade theta source estimation resulted in a similar estimate – peak activation MNI coordinates {*X* = 15; *Y* = 0; *Z* = 45}, matching the same brain region.

Post-keypress ERP neuronal source analysis identified the {*X* = −5; *Y* = −25; *Z* = 30} coordinates as the maximum peak of the resulting PSD map at around 28 ms after response beginning. These coordinates were estimated to match the Brodmann area 23 and possibly part of the posterior cingulate cortex (PCC) or a posterior part of ACC. Post-saccade ERP source analysis provided similar results for the time frames around 59 ms after response beginning. Peak activation MNI coordinates {*X* = 5; *Y* = 5; *Z* = 35}, estimated to match the Brodmann area 24 and, thus, possibly part of the ACC.

## Discussion

Here, we studied the EEG midfrontal theta modulation during performance monitoring for motor limb (hand) and oculomotor actions (keypress and saccade, respectively) in response preparation and execution. We aimed to scrutinize whether midfrontal theta modulation related to performance monitoring varies depending on the type of response in the moments before and after an error is committed. A go/no-go paradigm combining two types of instructions (facial cues and object cues) was used to investigate the neuronal mechanisms leading to erroneous actions and the ones related to the implementation of behavioral adjustments once an error is detected.

We started by analyzing action-related behavioral features to understand the differences between keypress and saccadic actions regarding reaction time and task performance. Then, we investigated the EEG signals recorded during response preparation and execution to map performance monitoring patterns. Our goal was to test how early performance monitoring is reflected by midfrontal theta power by comparing pre- and post-response patterns and test whether these dynamics were dependent or not on the type of action performed.

### Action Performance Variability

Our behavioral data analysis revealed differences in reaction time, number, and type of errors committed depending on the action performed (keypress or saccade). Moreover, it suggested a high number of errors related to action selection once we found many erroneous responses due to incorrect type of action performed or both keypress and saccade performed simultaneously. In particular, we found a high number of saccades occurring during keypress trials, which suggests participants’ difficulty in inhibiting saccadic actions during keypress trials. It also highlights higher impulsivity during saccadic responses relative to the keypress ones, which is supported by the shorter reaction times recorded. These suggestions are in line with the link between oculomotor actions and visual-based impulsive reaction pathways (Frens and Erkelens, 1991).

The absence of premature keypress, whereas a considerable number of premature responses occurred during saccadic trials, is also in agreement with such an assumption. Moreover, the most common error during keypress trials occurred by omission (no-go instead of go), whereas during saccadic trials occurred by commission of a pro-saccade instead of an anti-saccade or the opposite case. This might suggest that participants tended to easily inhibit keypress responses when they were not certain of the correct direction to go, particularly when comparing with the oculomotor ones.

Reaction time analysis revealed that correct responses tend to occur earlier than the erroneous ones, both for keypress and saccades, but by a wider difference for the keypresses. Previous studies have reported slight hesitations occurring for erroneous responses compared to the correct ones (Cavanagh et al., 2010), which might explain our results. Being a more controlled type of action, keypresses probably reflect more hesitation due to doubts associated with errors.

### Event-Related Potentials

When comparing the neuronal patterns during error commission and correct responses, we found an evident ERN in the midfrontal channels for both keypresses and saccades (and for erroneous responses mixing both actions), confirming the paradigm’s robustness to study error monitoring (Falkenstein et al., 2000; Cavanagh et al., 2012; Pavone et al., 2016).

The ERN pattern recorded for both response types was similar in amplitude, but with saccades leading to a slightly later ERN. On the one hand, this latency difference might be related to the recording method itself – while a keypress is recorded as an instantaneous action, the saccade is recorded as a movement with a higher duration variability (Gibaldi and Sabatini, 2021). On the other hand, the impulsivity reflected by shorter saccadic reaction time might be, here, represented by a later error perception, thus resulting in a later ERN latency. Participants might take a few milliseconds longer to perceive an incorrect saccadic movement. There is previous evidence that these error patterns are related to action-outcome interpretation (Falkenstein et al., 2000; Cavanagh et al., 2012; Pavone et al., 2016) and not the action itself, although this conclusion is still not consensual. Additionally, for some channels, for example, for FCz, the ERN amplitude seems, on average, larger for keypress errors than for saccade ones. Again, this might be related to a clearer perception of action-outcome or more synchronized response timing. Nevertheless, contrarily to the latency differences found for all tested midfrontal channels, this interaction between ERN amplitude and type of response was only found for FCz and FC2 channels. Therefore, even though there are ERN latency differences, our findings seem to suggest similar error-monitoring processes during both types of action.

Source analysis also suggested a similar neuronal origin for the ERN resulting from keypress and saccade errors. The ERN activity was estimated to originate from the Brodmann area 23 and 24 for keypress and saccade actions, respectively. These areas possibly match the anterior and PCC, already suggested by several previous studies as the ERN neuronal source (Olvet and Hajcak, 2008; Iannaccone et al., 2015; Pavone et al., 2016). The slight difference between both estimations might be justified by the structural proximity between these regions and the low spatial resolution of the estimation method. Previous studies also reported that the ERN can be generated from posterior regions of the ACC that directly connect with the PCC (O’Connell et al., 2007).

### Opposite Theta Modulation Patterns Preceding and Following Responses

Our results revealed an opposite midfrontal theta power modulation in the pre- and post-response moments depending on performance (error vs. correct) and independent of the response type (keypress vs. saccade). On the one hand, during response preparation, we found decreased theta activity for pre-error and when compared to pre-correct actions. On the other hand, we found increased theta activity during error commission and when compared to correct responses.

Our post-response results are in line with the assumption of midfrontal theta activity as a neuronal mechanism by which goal-relevant behavioral adjustments are implemented. Increased post-error theta has been reported to index adaptive adjustments required for the ongoing regulation of action (Nigbur et al., 2011) and to reflect a common computation used for realizing the need for cognitive control (Cavanagh and Frank, 2014). It has also been shown to be related to a wide range of conflict management processes, including the one resulting from an unexpected action-outcome (Cohen and Cavanagh, 2011; Nigbur et al., 2011; Vissers et al., 2018; Kaiser et al., 2019).

Regarding pre-response midfrontal theta, although there is no consensus about its functional meaning, previous studies have found increased theta to be predictive of an efficient response. Decreased pre-response theta has been suggested to underly diminished levels of attention (Atchley et al., 2017). Besides, increased theta power during response preparation has been associated with more efficient information interpretation and memory encoding (White et al., 2013; Wallis et al., 2015; de Vries et al., 2020). These oscillations may also facilitate coordination of neural processing in the sensorimotor pathways of the brain to support efficient decision-making. According to Cohen and Donner (2013), theta-band oscillatory synchronization is a mechanism by which information can be integrated over large-scale brain networks, thus predicting performance on cognitive control tasks.

Moreover, contrarily to the classical theory of midfrontal theta as a general domain-independent mechanism for cognitive control (Cavanagh et al., 2012; Cavanagh and Frank, 2014) domain-specific effects of theta power and connectivity, already during response preparation, have been demonstrated (Zuure et al., 2020; Kaiser and Schütz-Bosbach, 2021). Our study adds to this discussion by testing for different task dependencies. Our results did not reveal differences on midfrontal theta dependent on the action performed but did reveal different midfrontal patterns depending on distinct cognitive control mechanisms occurring in pre- and post-response moments.

The arguable involvement of visual sensory integration and perception to correctly interpret the facial expression, gazing, and object *silhouette* has been also reported to elicit midfrontal activation of monitoring networks (Labrenz et al., 2012; Chmielewski et al., 2014). As the proposed experimental paradigm required participants to correctly retain visual information, even if for short periods, we believe that poorly interpreted information and attention lapses influenced task performance and were reflected by decreased theta power preceding commission errors.

Additionally, the analysis of object-based instruction performance – evaluated based on incorrect type of action selection (or both actions simultaneously performed) – revealed the presence of significant ERN and error-related theta activity over the midfrontal channels following erroneous responses, and a significant decreased theta power in the moments preceding errors. This analysis provided us with a greater number of erroneous samples and prevented the inclusion of error potentials in the facial-related correct sample, thus contributing to the robustness of our findings.

According to our data, pre- and post-error midfrontal theta can be discriminated based not only on its modulation pattern but also on its neuronal source. The salience network, primarily composed of the ACC and the anterior insula, has been suggested to have a major role in self-control processes and self-awareness (Craig, 2002; Saper, 2002; Critchley, 2005; Heimer and van Hoesen, 2006; Seeley et al., 2007). Our findings suggest that midfrontal theta activity recorded in the moments following response might originate from the ACC, which is believed to function as an efferent hub from the salience network and be involved in the generation of cognitive, behavioral, and physiological responses (Seeley, 2019). It has been shown that this brain region is related to increased theta power (Cavanagh and Frank, 2014) and error signaling ERN (Gehring et al., 2012), leading to error perception and behavioral adjustment (Cavanagh et al., 2010, 2012; Womelsdorf et al., 2010; Nigbur et al., 2011; Cavanagh and Frank, 2014; Kaiser et al., 2019).

Importantly, post-response source analyses suggested that the ERN and post-error theta signals originate from similar regions. This is in agreement with previous studies, which highlighted the ERN and midfrontal theta relationship and suggested ERN as in part originated by the ongoing increase in theta power that follows error commission (Trujillo and Allen, 2007). Moreover, considering the phase shift between keypress and saccade ERN in our data, this suggestion is also in line with the later increase in post-saccadic error theta power, when compared with the post-keypress error theta power.

Our findings also suggest that the anterior insula, which has been shown to function as an afferent hub from the salience network and to be responsible for processing feedback originating from interconnected networks (Seeley, 2019), might be the source of the pre-response midfrontal theta. Some previous studies have proposed the anterior insula cortex to provide an early cognitive control signal in performance monitoring processes (Sridharan et al., 2008; Menon and Uddin, 2010; Ham et al., 2013; Bastin et al., 2017). It has been described as a driver of awareness, constantly receiving feedback information from interconnected networks (Craig, 2009), as the ventral attentional network, which is functionally related to error awareness (Klein et al., 2013). Thus, the link between a decreased theta activity and a greater probability of erroneous outcomes reported both in our data and in previous studies (Cohen and Donner, 2013; Atchley et al., 2017; van Noordt et al., 2017; Kaiser and Schütz-Bosbach, 2021), might be explained by the reduced feedback from different networks to the anterior insula. It possibly highlights diminished attention and poor coordination of neural processing required for an efficient response (White et al., 2013; Wallis et al., 2015; de Vries et al., 2020).

## Conclusion

The results of our study suggest an action-independent midfrontal theta role in performance monitoring. Its modulation patterns seem to alternate between attentional and correctional alert mechanisms for pre-response and post-response moments, respectively. Theta activity is inversely modulated in those two contexts, comparing correct and erroneous actions. Such patterns were found for two types of action – keypress and saccade – and two types of errors – errors within the same type of action (for example, a pro-keypress instead of an anti-keypress) and errors related to wrong action selection (for example, a keypress instead of a saccade).

Our results support previous evidence of midfrontal theta as an error prediction neuronal marker, opening several possibilities for automatic performance-related pattern classification. Moreover, they encourage further studies on how midfrontal theta reflects performance monitoring and salience network dynamics.

## Data Availability Statement

The raw data supporting the conclusions of this article will be made available by the authors, without undue reservation.

## Ethics Statement

The studies involving human participants were reviewed and approved by the Ethics Committee of the Faculty of Medicine of the University of Coimbra. The patients/participants provided their written informed consent to participate in this study.

## Author Contributions

JE, TS, and MC-B contributed to analysis and interpretation of the data. JE, CD, and DC contributed to data acquisition. All authors contributed to writing of the manuscript and study concept/design.

## Conflict of Interest

The authors declare that the research was conducted in the absence of any commercial or financial relationships that could be construed as a potential conflict of interest.

## Publisher’s Note

All claims expressed in this article are solely those of the authors and do not necessarily represent those of their affiliated organizations, or those of the publisher, the editors and the reviewers. Any product that may be evaluated in this article, or claim that may be made by its manufacturer, is not guaranteed or endorsed by the publisher.

## Supplementary Material

Supplementary Table 1 Facial-related instruction errors ERN latency (ms) estimates of fixed effects *per* channel using action (keypress vs. saccade) as factor and inter-subject variability as random effects.

Supplementary Table 2 Facial-related instruction performance ERP amplitude (μV) tests of fixed effects *per* channel using performance (correct vs. error), action (keypress vs. saccade), and their interaction as factors, and inter-subject variability as random effects.

Supplementary Table 3 Facial-related instruction performance ERN amplitude (μV) estimates of fixed effects *per* channel using performance (correct vs. error) as factor and inter-subject variability as random effects. Channels FC2 and FCz, estimates were analyzed separately for keypress (KP) and saccade (S) given that the respective tests of fixed effects revealed a significant performance vs. action interaction (see Supplementary Table 2).

Supplementary Table 4 Facial-related instruction performance theta power (dB) tests of fixed effects *per* channel using performance (correct vs. error), action (keypress vs. saccade), time (pre vs. post), performance vs. action interaction, performance vs. times interaction, and action vs. time interaction as factors and inter-subject variability as random effects.

Supplementary Table 5 Facial-related instruction performance theta power (dB) estimates of fixed effects *per* channel for the facial-related instruction performance using performance (correct vs. error) and action as factors and inter-subject variability as random effects. Channel FC1 was excluded from this analysis given that it showed no significant performance vs. time interaction in the tests of fixed effects (Supplementary Table 4).

Supplementary Table 6 Object-related instruction performance theta power (dB) tests of fixed effects *per* channel using performance (correct vs. error), time (pre vs. post), and performance vs. times interaction as factors and inter-subject variability as random effects.
