# Supplementary material for: An Action-Independent Role for Midfrontal Theta Activity Prior to Error Commission
Source: Front Hum Neurosci. 2022 May 11;16:805080. doi: 10.3389/fnhum.2022.805080 (PMC9131421; doi:10.3389/fnhum.2022.805080)
Supplement: Supplementary Table 1 — Facial-related instruction errors ERN latency (ms) estimates of fixed effects per channel using action (keypress vs. saccade) as factor and inter-subject variability as random effects. [file Table_1.pdf]

Channel	Keypress *vs* Saccade ERN Latency Linear Mixed Model Statistics		
	ERN Latency (ms)		
	Estimate (SD)	***t* value *(df)***	***p* value**
**F1**	-15.402 (2.546)	-6.046 (623.635)	< 0.001
**F2**	-16.348 (2.549)	-6.412 (623.603)	< 0.001
**Fz**	-13.338 (2.596)	-5.138 (631.000)	< 0.001
**FC1**	-19.693 (2.514)	-7.833 (626.026)	< 0.001
**FC2**	-15.329 (2.527)	-6.066 (623.475)	< 0.001
**FCz**	-17.389 (2.549)	-6.822 (620.532)	< 0.001

SD: *Standard deviation;* ***df:*** *Degrees of freedom*
