# Supplementary material for: An Action-Independent Role for Midfrontal Theta Activity Prior to Error Commission
Source: Front Hum Neurosci. 2022 May 11;16:805080. doi: 10.3389/fnhum.2022.805080 (PMC9131421; doi:10.3389/fnhum.2022.805080)
Supplement: Supplementary Table 2 — Facial-related instruction performance ERP amplitude (μV) tests of fixed effects per channel using performance (correct vs. error), action (keypress vs. saccade), and their interaction as factors, and inter-subject variability as random effects. [file Table_2.pdf]

Facial-based Instruction Performance – ERP Amplitude (μV) Linear Mixed Model Statistics				
**Channel**	***Test of Fixed Effects***			
	Factor	***F***	***df***	***p* value**
**F1**	***Performance***	15.838	4899.922	< 0.001
	Action	1.706	4883.165	0.192
	Performance vs Action	1.407	4883.346	0.236
**F2**	***Performance***	7.668	4899.253	0.006
	Action	6.393	4883.483	0.011
	Performance vs Action	0.180	4883.717	0.671
**Fz**	***Performance***	11.168	4899.959	0.001
	Action	1.072	4883.228	0.300
	Performance vs Action	1.066	4883.412	0.302
**FC1**	***Performance***	26.880	4899.829	< 0.001
	Action	1.642	4883.474	0.200
	Performance vs Action	1.596	4883.685	0.206
**FC2**	***Performance***	26.721	4883.947	< 0.001
	Action	8.934	4884.490	0.003
	Performance vs Action	4.239	4884.865	0.040
**FCz**	***Performance***	42.350	4899.559	< 0.001
	Action	0.707	4883.599	0.401
	Performance vs Action	7.144	4883.821	0.008

df: *Degrees of freedom*
