# Supplementary material for: An Action-Independent Role for Midfrontal Theta Activity Prior to Error Commission
Source: Front Hum Neurosci. 2022 May 11;16:805080. doi: 10.3389/fnhum.2022.805080 (PMC9131421; doi:10.3389/fnhum.2022.805080)
Supplement: Supplementary Table 3 — Facial-related instruction performance ERN amplitude (μV) estimates of fixed effects per channel using performance (correct vs. error) as factor and inter-subject variability as random effects. Channels FC2 and FCz, estimates were analyzed separately for keypress (KP) and saccade (S) given that the respective tests of fixed effects revealed a significant performance vs. action interaction (see Supplementary Table 2). [file Table_3.pdf]

Channel	Facial-based Instruction Performance – ERP Amplitude (μV) Linear Mixed Model Statistics			
	**Estimates of Fixed Effects**			
		Estimate (SD)	***t* value *(df)***	***p* value**
**F1**		0.778 (0.307)	2.535 (4896.258)	0.011
**F2**		0.616 (0.293)	2.103 (4898.392)	0.035
**Fz**		0.672 (0.320)	2.098 (4896.436)	0.036
**FC1**		1.014 (0.286)	3.541 (4897.675)	< 0.001
**FC2**	KP	1.842 (0.369)	4.987 (1718.233)	< 0.001
	S	0.666 (0.393)	2.633 (2817.882)	0.008
**FCz**	KP	2.491 (0.426)	5.845 (1966.395)	< 0.001
	S	0.909 (0.285)	3.192 (2816.937)	0.001

SD: *Standard deviation;* ***df:*** *Degrees of freedom*
