# Supplementary material for: An Action-Independent Role for Midfrontal Theta Activity Prior to Error Commission
Source: Front Hum Neurosci. 2022 May 11;16:805080. doi: 10.3389/fnhum.2022.805080 (PMC9131421; doi:10.3389/fnhum.2022.805080)
Supplement: Supplementary Table 4 — Facial-related instruction performance theta power (dB) tests of fixed effects per channel using performance (correct vs. error), action (keypress vs. saccade), time (pre vs. post), performance vs. action interaction, performance vs. times interaction, and action vs. time interaction as factors and inter-subject variability as random effects. [file Table_4.pdf]

Facial-based Instruction Performance – Theta Power (dB) Linear Mixed Model Statistics				
Channel	Pre and Post-response Test of Fixed Effects			
	Factor	***F***	***df***	***p* value**
**F1**	***Performance***	0.447	9408.444	0.504
	Action	0.107	9413.702	0.743
	Time	3.585	9415.186	0.058
	Performance vs Action	0.281	9416.203	0.596
	Performance vs Time	25.581	9413.098	< 0.001
	Action vs Time	5.161	9417.528	0.023
**F2**	***Performance***	6.058	9347.132	0.014
	Action	0.064	9415.111	0.800
	Time	0.691	9417.227	0.406
	Performance vs Action	1.747	9418.718	0.186
	Performance vs Time	18.516	9414.201	< 0.001
	Action vs Time	1.390	9420.510	0.238
**Fz**	***Performance***	1.990	9343.822	0.158
	Action	0.128	9413.168	0.721
	Time	1.427	9415.292	0.232
	Performance vs Action	0.099	9416.788	0.753
	Performance vs Time	20.185	9412.256	< 0.001
	Action vs Time	4.594	9418.582	0.032
**FC1**	***Performance***	0.097	7759.274	0.756
	Action	1.986	9418.706	0.159
	Time	0.213	9423.580	0.645
	Performance vs Action	0.133	9427.792	0.716
	Performance vs Time	3.456	9415.352	0.063
	Action vs Time	0.016	9430.025	0.900
**FC2**	***Performance***	6.379	9368.506	0.012
	Action	0.035	9414.139	0.851
	Time	2.776	9416.066	0.096
	Performance vs Action	0.416	9417.412	0.519
	Performance vs Time	18.673	9413.324	< 0.001
	Action vs Time	2.480	9419.071	0.115
**FCz**	***Performance***	0.259	9302.540	0.611
	Action	2.398	9415.258	0.122
	Time	0.000	9417.662	0.985
	Performance vs Action	0.948	9419.380	0.330
	Performance vs Time	20.609	9414.195	< 0.001
	Action vs Time	0.537	9421.363	0.464

df: *Degrees of freedom*
