# Supplementary material for: An Action-Independent Role for Midfrontal Theta Activity Prior to Error Commission
Source: Front Hum Neurosci. 2022 May 11;16:805080. doi: 10.3389/fnhum.2022.805080 (PMC9131421; doi:10.3389/fnhum.2022.805080)
Supplement: Supplementary Table 5 — Facial-related instruction performance theta power (dB) estimates of fixed effects per channel for the facial-related instruction performance using performance (correct vs. error) and action as factors and inter-subject variability as random effects. Channel FC1 was excluded from this analysis given that it showed no significant performance vs. time interaction in the tests of fixed effects (Supplementary Table 4). [file Table_5.pdf]

Channel	Facial-based Instruction Performance – Theta Power (dB) Linear Mixed Model Statistics					
	Pre and Post-response Estimates of Fixed Effects					
	**Pre-response**			**Post-response**		
	Estimate (SD)	***t value (df)***	***p* value**	***Estimate (SD)***	***t value (df)***	***p* value**
**F1**	0.666 (0.228)	2.921 (4458.323)	0.004	-0.767 (0.211)	-3.635 (4898.764)	< 0.001
**F2**	0.188 (0.230)	0.817 (4250.865)	0.414	-1.134 (0.181)	-6.260 (4899.371)	< 0.001
**Fz**	0.605 (0.262)	2.307 (4331.710)	0.021	-1.069 (0.254)	-4.210 (4897.950)	< 0.001
**FC1**	-	-	-	-	-	-
**FC2**	0.178 (0.133)	1.334 (4387.934)	0.182	-0.699 (0.132)	-5.283 (4899.992)	< 0.001
**FCz**	0.690 (0.302)	2.288 (4034.886)	0.022	-0.971 (0.215)	-4.522 (4899.504)	< 0.001

SD: *Standard deviation;* ***df:*** *Degrees of freedom*
