# Supplementary material for: An Action-Independent Role for Midfrontal Theta Activity Prior to Error Commission
Source: Front Hum Neurosci. 2022 May 11;16:805080. doi: 10.3389/fnhum.2022.805080 (PMC9131421; doi:10.3389/fnhum.2022.805080)
Supplement: Supplementary Table 6 — Object-related instruction performance theta power (dB) tests of fixed effects per channel using performance (correct vs. error), time (pre vs. post), and performance vs. times interaction as factors and inter-subject variability as random effects. [file Table_6.pdf]

Object-based Instruction Performance – Theta Power (dB) Linear Mixed Model Statistics				
Channel	Pre and Post-response Test of Fixed Effects			
	Factor	***F***	***df***	***p* value**
**F1**	***Performance***	0.035	7754.226	0.851
	Time	647.609	8263.977	< 0.001
	Performance vs Time	0.260	8250.970	0.610
**F2**	***Performance***	0.773	8113.800	0.379
	Time	287.403	8259.667	< 0.001
	Performance vs Time	3.694	8247.207	0.055
**Fz**	***Performance***	0.08	8081.48	0.778
	Time	611.097	8261.406	< 0.001
	Performance vs Time	3.279	8248.631	0.070
**FC1**	***Performance***	1.692	3147.344	0.193
	Time	288.577	8144.966	< 0.001
	Performance vs Time	1.479	8261.242	0.224
**FC2**	***Performance***	0.584	7011.231	0.445
	Time	25.42	8259.459	< 0.001
	Performance vs Time	1.492	8252.059	0.222
**FCz**	***Performance***	1.082	8163.039	0.298
	Time	146.943	8258.269	< 0.001
	Performance vs Time	13.263	8246.651	< 0.001

df: *Degrees of freedom*
